# Independent Mobility Achieved through a Wireless Brain-Machine Interface

**DOI:** 10.1371/journal.pone.0165773

**Published:** 2016-11-01

**Authors:** Camilo Libedinsky, Rosa So, Zhiming Xu, Toe K. Kyar, Duncun Ho, Clement Lim, Louiza Chan, Yuanwei Chua, Lei Yao, Jia Hao Cheong, Jung Hyup Lee, Kulkarni Vinayak Vishal, Yongxin Guo, Zhi Ning Chen, Lay K. Lim, Peng Li, Lei Liu, Xiaodan Zou, Kai K. Ang, Yuan Gao, Wai Hoe Ng, Boon Siew Han, Keefe Chng, Cuntai Guan, Minkyu Je, Shih-Cheng Yen

**Affiliations:** 1 Department of Psychology, National University of Singapore, Singapore, Singapore; 2 Singapore Institute for Neurotechnology, National University of Singapore, Singapore, Singapore; 3 Singapore Institute for Clinical Sciences, A*STAR, Singapore, Singapore; 4 Institute for Infocomm Research, A*STAR, Singapore, Singapore; 5 National Neuroscience Institute, Singapore, Singapore; 6 Institute of Microelectronics, A*STAR, Singapore, Singapore; 7 Department of Electrical and Computer Engineering, National University of Singapore, Singapore, Singapore; 8 School of Computer Science and Engineering, and School of Mechanical and Aerospace Engineering, Nanyang Technological University, Singapore, Singapore; Wadsworth Center, UNITED STATES

## Abstract

Individuals with tetraplegia lack independent mobility, making them highly dependent on others to move from one place to another. Here, we describe how two macaques were able to use a wireless integrated system to control a robotic platform, over which they were sitting, to achieve independent mobility using the neuronal activity in their motor cortices. The activity of populations of single neurons was recorded using multiple electrode arrays implanted in the arm region of primary motor cortex, and decoded to achieve brain control of the platform. We found that free-running brain control of the platform (which was not equipped with any machine intelligence) was fast and accurate, resembling the performance achieved using joystick control. The decoding algorithms can be trained in the absence of joystick movements, as would be required for use by tetraplegic individuals, demonstrating that the non-human primate model is a good pre-clinical model for developing such a cortically-controlled movement prosthetic. Interestingly, we found that the response properties of some neurons differed greatly depending on the mode of control (joystick or brain control), suggesting different roles for these neurons in encoding movement intention and movement execution. These results demonstrate that independent mobility can be achieved without first training on prescribed motor movements, opening the door for the implementation of this technology in persons with tetraplegia.

## 1. Introduction

One of the main obstacles faced by tetraplegic persons is the lack of independent mobility [1]. A promising new tool that may help mitigate this problem is a brain–machine interface (BMI), where brain signals are used to directly control a machine, thus bypassing the damaged tissue in the spinal cord. BMI wheelchair control systems have been developed using non-invasive methods to measure brain activity, such as electroencephalography (EEG) [2–4]. While EEG-based BMI systems enjoy the benefit of being non-invasive, their usefulness to individuals remains limited due to the low signal-to-noise ratio and poor spatial resolution of EEG. For example, some EEG-based BMI implementations are slow, with delays in the order of several seconds [2, 4], or require significant amounts of machine intelligence to guide wheelchair movements [3]. In contrast, invasive techniques, such as microelectrode recordings, have higher spatial resolution and signal-to-noise ratio, making them a viable alternative to non-invasive methods. Activity of single neurons in motor cortex has been used to control cursors on computers [5, 6], as well as to control virtual, robotic [7–11] and real arms through muscle stimulation [12]. Furthermore, for a BMI system to be used by a person, it is necessary to train the decoding algorithm without having the subject perform a specific motor task (e.g. moving a joystick), which paralyzed individuals cannot perform. We implemented this approach by initially training the monkeys to control the robotic platform using brain signals for 11 months, using algorithms trained from overt movements. After this period, we were confident that the animals understood that they could control the platform using their intention to move, without actually moving a joystick. We then trained all subsequent decoders in the absence of a joystick and overt movements, and demonstrated that performance of these decoders was comparable with those trained with overt movements. We showed that two monkeys were able to control a robotic platform with no machine intelligence in a free-running, fast and accurate manner, resembling the performance they achieved using joystick control.

### One Sentence Summary

Macaques used a wireless intracortical brain-machine interface to control a robotic platform to achieve independent mobility.

## 2. Materials and Methods

**Fig 1:**
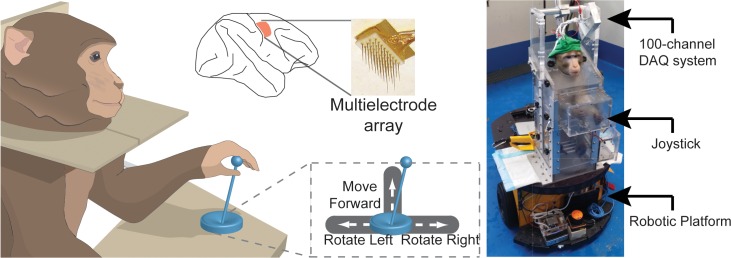
Setup Description and Electrode Locations. Animals were trained to move a robotic platform (right) using a joystick. The joystick was spring-loaded, returning to the center position when released. Movement of the joystick was restricted to left, right, and forward movements, while not allowing for diagonal movements. The robotic platform rotated in place in the counter-clockwise and clockwise directions with left and right joystick movements, respectively, and moved forward with forward joystick movements (bottom-middle inset). Movement commands from the joystick reached the platform serially, so at any point in time only one command was executed. Joystick movements were translated to platform movements in discrete states, such that if the joystick moved past a threshold, the platform would move with a fixed speed after initial acceleration. Multiple microelectrode arrays were implanted in the arm and hand areas of primary motor cortex (top middle).

Two macaques (*Macaca Fascicularis*) were trained to move a robotic platform towards a goal positioned at different locations in a room using a 3-direction joystick (Fig 1). After training in this “Joystick Control” task, both animals were implanted with multiple electrode arrays in the hand/arm area of primary motor cortex (96 and 128 electrodes in Animal A and B, respectively, Fig 1). A custom-built microchip amplified, digitized, and wirelessly transmitted broadband neuronal signals to a computer in the primate chair (Fig 1). The animals were then trained to control the robotic platform using the single-unit activity recorded from the implanted electrodes, either in the presence of a joystick (“BMI Control with Joystick”), or without the joystick (“BMI Control without Joystick”). Brain signals were used to determine movement initiation and direction in real-time, which allowed the animals to move around on the robotic platform in a free-running mode. Importantly, no machine intelligence was used to guide the movement of the platform (S1 Movie).

### 2.1 Electrode implantation and animal care

We used two male adult macaques (*Macaca fascicularis*), Animal A and Animal B, in the experiments. All animal procedures were approved by, and conducted in compliance with the standards of the Agri-Food and Veterinary Authority of Singapore and the Singapore Health Services Institutional Animal Care and Use Committee (Singhealth IACUC #2012/SHS/757). The procedures also conformed to the recommendations described in Guidelines for the Care and Use of Mammals in Neuroscience and Behavioral Research (National Academies Press, 2003). Each animal was implanted first with a titanium head-post (Crist Instruments, MD, USA) before arrays of intracortical microelectrodes (MicroProbes, MD, USA) were implanted in the hand/arm region of the left primary motor cortex (Fig 1, middle top). In Animal A, we implanted 2 arrays of 16 electrodes each and 2 arrays of 32 electrodes each, for a total of 96 electrodes. In Animal B, we implanted 4 arrays of 32 electrodes each, for a total of 128 electrodes. The arrays consisted of platinum-iridium wires with 400 μm separation, 1–1.5 mm of length, 0.5 MΩ of impedance, and arranged in 4x4 or 8x4 grids.

### 2.2 Signal acquisition, processing and spike detection

Spike signals were acquired using an in-house 100-channel wireless neural recording system [13–15] which sampled neural data at 13 kHz.

The wide-band signals were band-pass filtered between 300 to 3000 Hz to remove low frequency components. Spikes were detected using an automated threshold-crossing criterion selected for each channel. The threshold (Thr) for spike detection was found using the formula [16]:

Thr=5σ;σ=median{|x|/0.6745}

where x is the filtered signal, and σ is an estimate of the standard deviation of the background noise. The number of units typically isolated each day was 40 (33 to 51) in both animals. All recorded units were used for BMI Control.

### 2.3 Behavioral tasks

Animals were trained on two tasks: (1) Single-movement task, where the animal was required to follow one of four commands on each trial to reach a target: turn right for 90°, turn left for 90°, move forward for 2 m, or stay still for at least 5 s (Right, Left, Forward, Stop). Motion in one direction was sufficient to reach the target. The target was a trainer holding a reward, standing 2 m in front, or directly to the right or left of the monkey. A sound cue signaled the ‘Stop’ trials. A trial was considered successful if the monkey reached a ‘Left’, ‘Right’, or ‘Forward’ target within 15 s, or stayed completely still for 5 s during a ‘Stop’ trial; and (2) Free-movement task, where the animals moved freely in an asynchronous manner with no trial structure to reach the target (a trainer holding a reward, who moved around the room).

During the single-movement task, once the animal reached the target location, the control of the platform was turned off to prevent the platform from moving while the trainer was rewarding the animal. The control of the platform was then turned back on at the start of another trial. During the free-movement task, the animals were given full continuous control of the platform.

For each session of data collection for the single-movement task, we alternated, at random, blocks of Joystick Control, BMI Control with Joystick, and BMI Control without Joystick (60 trials of the same type per block). We repeated this alternation as long as the animal was willing to continue working (between 2–3 blocks; around 2 hours).

### 2.4 Animal training and decoder description

Both animals were trained to control the platform using a joystick (Joystick Control). Forward joystick movement would produce a forward translation of the platform, while left and right joystick movements would produce a leftward (counter-clockwise) and rightward (clockwise) rotation of the platform, without translation.

To allow the animals to get used to controlling the robotic platform using only cortical activity, we implemented a series of intermediate steps. First, we kept the joystick in place, but disconnected it from the control of the platform (BMI Control with Joystick), and decoded the animals’ brain activity using a model trained with Joystick Control trials (“*Initial Decoder*”). With this procedure, the animals were able to continue moving the dummy joystick, and did not have much difficulty with the task. Next, we removed the joystick from the platform, and the animals were required to control the platform using brain signals without the help of a dummy (BMI Control without Joystick). This proved challenging for the animals, and they frequently became frustrated at their inability to control the platform. Thus, we implemented an assisted control paradigm by correcting 50% of the decoder’s output to match the direction of the target. Using neural signals collected during these trials, we re-trained the decoder (‘*Recalibrated Decoder*”) and both animals were able to successfully perform the task afterwards.

After the monkeys were trained for 11 months, and had mastered BMI Control without Joystick using the *Recalibrated Decoder*, we started initiating experimental sessions by training the decoder directly using signals collected while the monkey performed BMI Control without Joystick, starting with a randomly initialized model and a 90% correction rate. Neural activity collected during these trials was then used to train a second decoder (“*Randomly Initialized Decoder*”) in the same manner as described above for the *Recalibrated Decoder*.

### 2.5 Decoding algorithm and models

A four-class assisted-learning classifier using linear discriminant analysis (LDA) was used for all decoding. The steps for linear discriminant analysis can be summarized as follows:

1. The mean firing rates for all simultaneously recorded neurons for the four different classes were computed to create a mean vector for each class.
2. Between-class and within-class scatter matrices were computed.
3. The eigenvectors for the scatter matrices were computed.
4. The eigenvector matrix was then used to transform incoming firing rates onto the new subspace for classification.

For individual neurons, firing rates were computed using a 500 ms window, which moved in 100 ms steps. Firing rates were calculated as the number of detected spikes divided by the length of the time window. Each decoder was trained using the firing rates of all the cells recorded simultaneously during 20 successful trials of single-movement tasks (5 trials and at least 250 windows in each direction).

The *Initial Decoder* was trained using neuronal signals collected while the animals performed Joystick Control. Firing rates during the execution of Leftward, Rightward, Forward, or Stop joystick movements during each trial were grouped to form the training data for the 4 movement classes in the model.

For the *Recalibrated Decoder*, neuronal signals were collected while the animals performed BMI Control using outputs from the *Initial Decoder*, but without the joystick and with a 50% correction rate. Specifically, neuronal responses were grouped according to the target location from each trial as the training data for the *Recalibrated Decoder*. These responses were included regardless of whether the decoded directions from the *Initial Decoder* were the same as the target directions. An implicit assumption underlying this approach is that the monkeys’ intentions were always to move towards the target during each trial.

For the *Randomly Initialized Decoder*, an initial training phase consisted of a randomly initialized decoder with 90% assistance. Adding 10% randomness introduced error trials, which provided a closer resemblance to what the animals would experience when controlling with the *Recalibrated Decoder*. In a subsequent phase, we retrained the decoder using 50% assistance.

Decoding was implemented in Matlab (Mathworks Inc, Massachusetts, USA) running on a computer in the robotic platform. The delay between neuronal signal acquisition and decoder output (including reading the neuronal signals from the data file, filtering, and spike detection) was, on average, 45 ms. Decoding occurred every 100 ms during BMI control of the platform.

### 2.6 Robotic Platform and Control Software

We used a commercial robotic platform (Adept MobileRobots LLC, USA). The mode of operation and degrees of freedom resembled that of commercial motorized wheelchairs (e.g. Drive Medical Design and Manufacturing). The platform used 2 motors to control translation and rotation. Command updates were sent from a computer inside the platform every 100 ms. The robotic platform had a latency of 12 ms for movement initiation, and accelerated from rest to a full-speed of 0.5 m/s in 3 s. We implemented a safety feature onboard the platform to avoid direct collision with objects. To this end, we equipped the robotic platform with 2 lasers (one in the front and one in the back) that would detect object proximity. When objects were detected at a distance of 56 cm from the platform, the speed was reduced linearly until it came to a stop when objects were 18 cm away.

### 2.7 Data analysis

**Fig 2:**
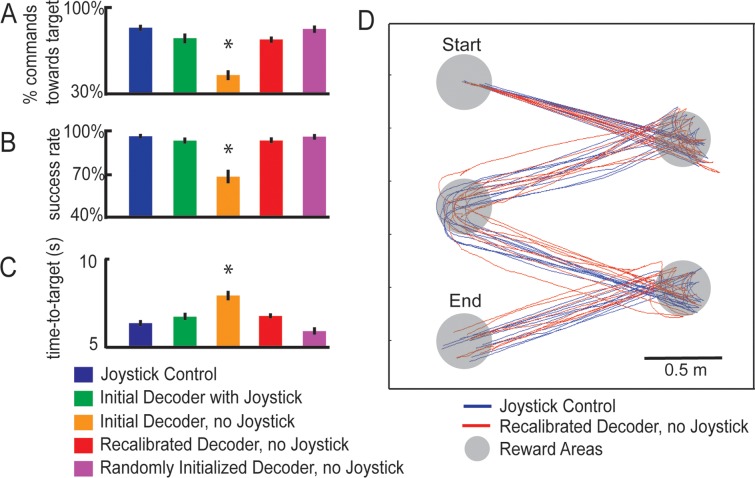
Performance under different modes of control. (A) Accuracy of decoder, defined as the proportion of decoded directions that matched the target location, in the single-movement task (chance performance 25%). (B) Success rate, defined as the percentage of trials in which the animals reached the reward location within 15 seconds, in the single-movement task (chance performance ~0%). (C) Average time that the animals took to reach the target during correct trials in the single-movement task. Error bars represent the standard error of the mean, and asterisks denote results that were significantly different from those of the Joystick Control task (blue bars, t-test, p<0.01). (D) Platform trajectories during Joystick Control (blue lines) and BMI Control using the *Recalibrated Decoder* (red lines) in the free-movement task that required the monkeys to move sequentially through a series of targets. The gray circles represent target locations. Animals controlled the platform continuously from the start until the end point. Trajectories were collected during a single experimental session.

All quantification of behavioral performance, decoding accuracy, and neural responses were performed using data collected during experimental sessions involving single-movement tasks. To compare behavioral performance between the various platform control modes, we calculated the percentage of successful trials, in which the monkey reached the target location under 15 s, as well as the time taken to reach the targets for each trial. For this measurement the level of chance is close to zero, as you would need roughly 70% of decoded commands to be directed towards the target direction in order for the trial to be successful. Therefore, chance success would be roughly (0.25)^0.7*150^ x 100 = (0.25)^105^ x 100 = 6.07x10^-62^ ≈ 0%. All values reported in Fig 2A are significantly different from chance (t-test < 0.01).

The accuracy of each decoder was determined by calculating the percentage of decoded commands that matched the movement required to reach the target in the single-movement task. To allow a fair comparison between Joystick Control, BMI Control with Joystick, and BMI Control without Joystick, the ground truth at every time step during each trial was defined as the target location for that trial. Decoding accuracy was calculated as the percentage of commands directed towards the target location (where the trainer was standing holding the reward). Chance performance in for decoding accuracy is 25%. All values reported in Fig 2A are significantly different from chance (t-test < 0.01).

Analysis of neural data was performed using a total of 182 cells recorded during 9 experimental sessions (4 in Animal A and 5 in Animal B). All 9 sessions were performed on different days. We compared neural activity when the animals performed Joystick Control to BMI Control without Joystick. We assumed that the cells recorded each day were unique, although it is possible that a subset of these cells corresponded to the same cells recorded in earlier sessions over multiple days. Should] Cells were categorized as selective based on neural activity 500–1500 ms after the trial-start cue (one-way ANOVA, p<0.01). For cells with selectivity during both Joystick and BMI Control, we performed a post-hoc analysis to determine whether the activity in the two modes of control differed. To this end, we compared neural firing during each of the 4 movement tasks (forward, left, right, stop) for Joystick control and BMI control using t-tests (p<0.01). If one or more of these tests showed significance, the cell was categorized as “Joystick-BMI (Different)”.

## 3. Results

### 3.1 Performance of Decoding Algorithms

We decoded neuronal signals using linear discriminant analysis. On each day, the firing rates of all recorded cells during the four types of joystick movements during Joystick Control (i.e. forward, left, right, and no movement) were used to form the training set for the Initial Decoder. The monkeys were able to successfully control the robotic platform using the *Initial Decoder* when the joystick was disconnected from the robotic platform but still present in the chair (BMI Control with Joystick) (Fig 2A–2C green bars, chance performance for decoding accuracy is 25%; chance performance for success rate ~0%; S2 Movie). It should be noted that during this mode of control, the monkeys were allowed to move the joystick, just like they did during Joystick Control, but the movement of the platform was controlled by the decoder instead of the joystick itself.

**Fig 3:**
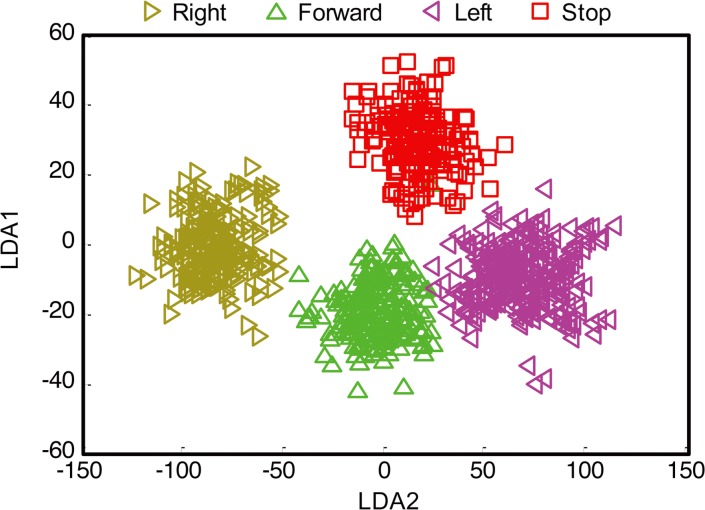
Linear discriminant space for a sample session. Projection of the firing rates onto two largest components of the linear space for the population of neurons collected during a sample BMI Control session (see detailed Methods).

Next, we attempted to train the monkeys to control the robotic platform using the *Initial Decoder* with the joystick removed from the chair. After one month of daily training sessions, neither monkey was able to successfully control the robotic platform (Fig 2A–2C orange bars, S1 Fig). During these sessions, we noticed significant differences in neuronal selectivity between Joystick Control and BMI Control without Joystick (described below), which prompted us to recalibrate the decoding model. We implemented a “*Recalibrated Decoder*” that was trained using signals collected while the animal performed BMI Control using the *Initial Decoder* [17], but with a 50% correction rate. We found that with this *Recalibrated Decoder*, monkeys were able to achieve accurate BMI Control (without correction) after a short calibration period of 10 to 15 minutes (Fig 2A–2C red bars, S1 Fig, S3 and S4 Movies). Furthermore, the trajectories followed by the platform in the free-moving task with multiple reward locations were similar for Joystick Control and *Recalibrated Decoder* control (Fig 2D, S2 Fig), indicating smooth control of the platform using this decoder. Firing rates collected during the second training stage yielded clear clusters within the linear discriminant space (Fig 3) that allowed subsequent decoding with high fidelity.

The above approach was used because the animals were initially trained to control the platform using a joystick and hence the *Initial Decoder* was trained using Joystick Control signals. However, a limitation of this approach is that it requires an initial training session using the joystick, which would not be possible in tetraplegic individuals [18]. In human subjects, the initial decoder is usually trained by instructing them to imagine the desired movements while neuronal activity in their motor cortices are collected [9]. Activity collected during neural control is then used to train and recalibrate the decoder [9–11]. We tested the feasibility of this approach using monkeys. After the monkeys became proficient in performing BMI Control without Joystick, we were able to train a *Randomly Initialized Decoder* directly without using the joystick at all. We found that with this strategy, monkeys were still able to achieve accurate BMI Control (without correction) after a short calibration period of 5 to 10 minutes (Fig 2A–2C purple bars, S1 Fig)[7, 8, 10]. We believe this is because the animals were highly familiar with the task, and despite the absence of the joystick, were mostly likely imagining the movements, just like the human subjects would be doing.

### 3.2 Response Properties of Neurons

**Fig 4:**
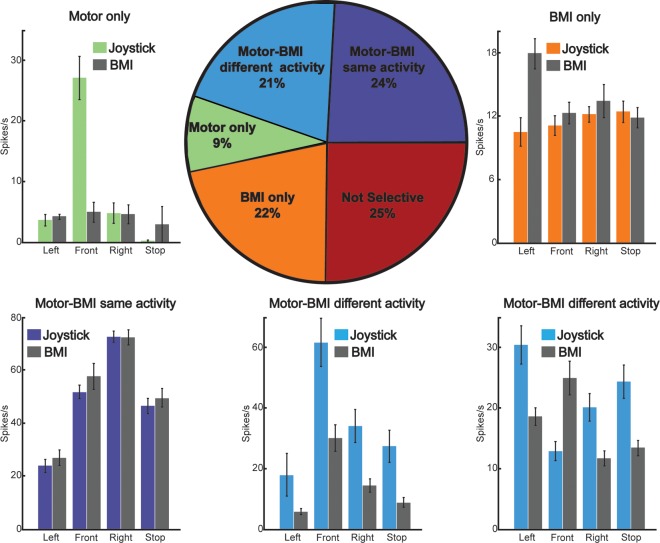
Neurons exhibited selectivity for movement direction and mode of control. **(**Pie chart) percentage of cells with different response profiles. Cells were categorized as selective based on the activity 500–1500 ms after the trial-start cue (left, forward, right, or stop; one-way ANOVA p<0.01). In our sample, 25% of cells showed no selectivity (red), 22% showed selectivity during BMI Control only (orange), and 9% showed selectivity during Joystick (motor) Control only (green). The rest of the cells (45%) showed selectivity during both modes of control. This last group was further subdivided into cells where activity during Joystick and BMI Control showed no significant differences (24%, purple) and cells where at least one category of movement was significantly different between Joystick and BMI Control (21%, light blue). (Bar plots) (A–E): Mean firing rates of example cells with different response profiles. Colored bars represent the activity during Joystick Control and gray bars during BMI Control. Error bars represent the standard error of the mean across trials.

Previous studies have shown that the tuning properties of neurons are different when comparing Joystick and BMI control [8, 11, 19]. Our data show that the majority of the neurons (75%) exhibited selective activation for the four different movement categories (left, front, right, or stop) during Joystick (Motor) Control, BMI Control (without joystick), or both (Fig 4, pie chart). Cells were categorized as belonging to one of four response groups: (1) “Motor-BMI (Same)” neurons (24% of cells) exhibited selective activation during both Joystick and BMI Control, but there was no significant difference in the activity between the two modes of control (Fig 4C); (2) “Motor-BMI (Different)” neurons (21% of cells) exhibited selective activation during both Joystick and BMI Control, but there were significant differences between the two modes of control in at least one of the four movement categories (Fig 4D and 4E); (3) “Motor-only” neurons (9% of cells) exhibited selective activation during Joystick Control only (Fig 4A); and (4) “BMI-only” neurons (22% of cells) exhibited selective activation during BMI Control only (Fig 4B). Of note, neurons in the “Motor-BMI (Different)” category had a variety of response profiles, such as cells with similar selectivity but overall reduction in firing rates (Fig 4D), and cells with similar overall firing rates, but with different selectivity (Fig 4E).

## 4. Discussion

We have shown in this study that macaques are able to achieve independent mobility using a wireless brain-machine interface. The performance of BMI control in our setup was not different from that achieved using joystick control, both in terms of direction of trajectories, and time to reach targets (Fig 2). This stands in contrast to the state-of-the-art EEG-based BMI technologies, which can take several seconds to execute a command [2, 4], and require extensive shared control with machine intelligence [3]. An additional key difference is that EEG-based BMI systems use coarse motor imagery to achieve control, such as imagining moving the left arm versus the right arm, whereas the spatial resolution of microelectrode recordings allowed us to use the much more natural activity that was related to the movement of one arm in different directions. The higher information bandwidth of microelectrode recordings has also been used to control a larger gamut of devices, such as controlling two robotic arms simultaneously [20].

An implicit assumption about this line of work is that the control of the robotic platform in a well-controlled laboratory setting can be generalized to everyday activities [7]. However, it is possible that the range of behaviors that can be implemented with BMI Control are bound by the restrictions imposed by the laboratory setting. During BMI sessions, we observed the emergence of untrained behaviors. For instance, one animal tended to direct the robotic platform towards the exit door whenever it was left open, regardless of where the treats where located (presumably to look at the animals housed in nearby cages) (S5 Movie). This behavior was not part of the task, but reflected the animals’ goals and preferences, highlighting that the robotic platform became a tool for the animals to exert willful independent mobility.

We have also shown that monkeys can use decoding algorithms trained in the absence of joystick movements to achieve BMI control of the robotic platform. The ability to accomplish this is important, since most of the technology development that precedes human trials generally takes place using the monkey model [7, 8, 11]. This approach in monkeys is difficult because it is hard to train monkeys to think about intended movements, without actual movements. We successfully accomplished this by training the monkeys for several months in a BMI task that used the *Recalibrated Decoder*. With this task they learned how to control the robotic platform using motor cortex activity in the absence of joystick movements. Once the monkeys’ performance reached levels comparable to those achieved using joystick control, we implemented the *Randomly Initialized Decoder* method. The control quality achieved using the *Randomly Initialized Decoder* resembled that achieved using joystick control (Fig 2), and the decoder can be calibrated efficiently, usually within 10 minutes. Of note, the *Randomly Initialized Decoder* did not involve the animal learning how to use a random decoder to control the platform. Rather, we used the random decoder as a starting point to train a second decoder (*Randomly Initialized Decoder*), which leveraged on the neurons’ existing selectivity while the animal was performing BMI control of the platform.

Finally, we characterized the response properties of neurons during Joystick and BMI Control and found important differences in the response properties between both modes of control. While some cells were selectively active only during Joystick Control or BMI Control, almost half of the cells recorded were selectively active during both modes of control, either with similar or different activations. Since neurons in the “Motor-BMI (Same)” group were active during both modes of control in a similar way, their activity appeared to reflect intended direction of movement, rather than a specific mode of control. On the other hand, the activity of neurons in the “Motor-only” category may be related to actual arm and hand movements in controlling the joystick. The activity of neurons in the “Motor-BMI (Different)” and “BMI-only” categories may reflect the use of a different strategy during BMI Control, compared to Joystick Control [17, 19], or may reflect the result of the learning process that the animals underwent to control the platform using BMI.

A limitation of this study is that we did not control for arm movements or muscle activation signals during BMI Control, and additionally, we did not block these signals using peripheral nerve blockers. Thus, although we were aiming for no overt movements in the monkey during BMI Control without Joystick, some residual movements were present, and it is possible that during BMI Control, we decoded motor signals associated with different movements than those executed during Joystick Control. The changes in neural activities observed during BMI Control without Joystick could also be related to these new forms of arm movements during BMI Control. These movements, however, may be part of a new strategy that the monkey is employing for BMI Control. This type of residual muscle activation could also constitute a control strategy for individuals with tetraplegia, some of whom have residual movement in their limbs or muscles in other parts of the body [7, 9]. For future designs of such studies, measures should be included to ensure that residual movements in the animals are kept to a minimum.

In this study, we have expanded the scope of neuron-based BMI Control through the use of platform command algorithms that enable smooth control of a robotic platform to enable independent mobility. These methods can be incorporated into future designs of BMI Control of robotic platforms, as well as to integrate the simultaneous control of robotic platforms and prosthetic arms [9].

## Supporting Information

S1 Fig Performance of different modes of control for each animal. (Top) Success rate, defined as the percentage of trials in which the animals reached the reward location within 15 seconds. (Bottom) Average time that animals took to reach the targets. Error bars represent the standard error of the mean.
(JPG)

S2 Fig Distance and angle travelled for different modes of control. (Left) Distance travelled and (Right) amount turned for joystick and BMI control. Trajectories were tracked using an automatic object detection algorithm applied to videos taken during the trials. Error bars represent the standard error of the mean. Asterisks denote a significant difference (ANOVA, p<0.05).
(JPG)

S1 Movie Wireless control of the robotic platform using brain-machine interface. Signals from the hand/arm area of primary motor cortex were wirelessly transmitted and decoded in real-time to control the movement of the robotic platform.
(MP4)

S2 Movie *Initial decoder* with joystick present, single movement task. The platform was controlled by the *Initial Decoder*, with the joystick still present for the animal to move. Joystick signals were not used to control the platform. The animal was performing the single-movement task, where motion in one direction was sufficient to reach the target. A double tone indicated a trial had started, and enabled platform movement. A single tone indicated the trial had ended, and disabled platform movement.
(MP4)

S3 Movie *Recalibrated Decoder* without joystick, single movement task. The platform was controlled by the *Recalibrated Decoder* in the absence of a joystick. The animal was performing the single-movement task, where motion in one direction was sufficient to reach the target. A double tone indicated a trial had started, and enabled platform movement. A single tone indicated the trial had ended.
(MP4)

S4 Movie *Recalibrated Decoder* without joystick, free movement task. The platform was controlled by the *Recalibrated Decoder* in the absence of a joystick. The animal was performing the free movement task, where the goal was to reach the trainer location and the platform was continuously under the monkeys’ control.
(MP4)

S5 Movie Untrained behaviors emerged during BMI Control, multi-movement task. The platform was controlled by the *Recalibrated Decoder* in the absence of a joystick. The animal was performing the free-movement task, where motion in more than one direction was required to reach the target. Left panel, curtain closed. Right panel, curtain open. When the curtain was closed, the animal ignored treats and moved towards nearby cages.
(MP4)
