# Supplementary figures and images for: Independent Mobility Achieved through a Wireless Brain-Machine Interface
Source: PLoS One. 2016 Nov 1;11(11):e0165773. doi: 10.1371/journal.pone.0165773 (PMC5089763; doi:10.1371/journal.pone.0165773)

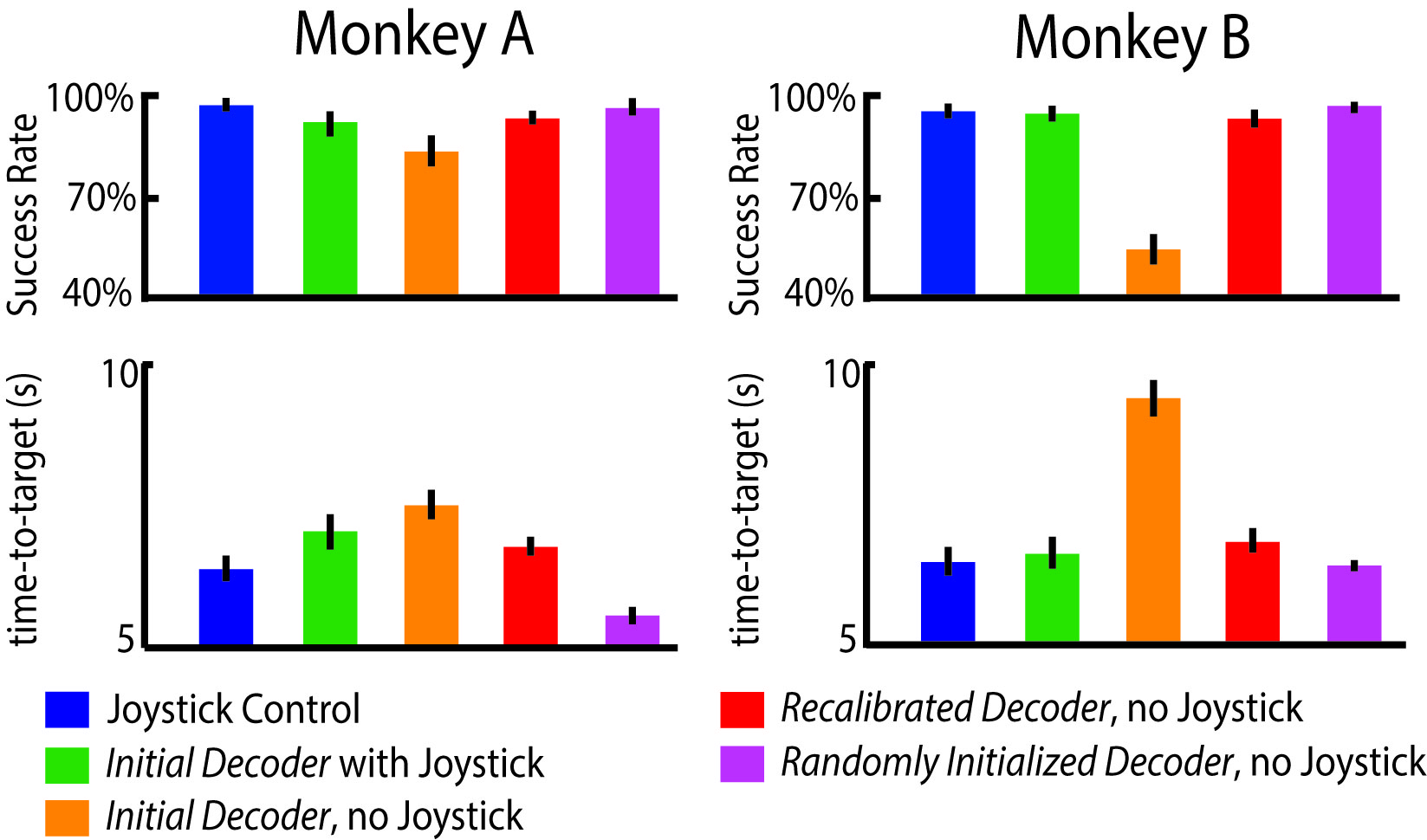

Supplement: S1 Fig — (Top) Success rate, defined as the percentage of trials in which the animals reached the reward location within 15 seconds. (Bottom) Average time that animals took to reach the targets. Error bars represent the standard error of the mean. (JPG) [file pone.0165773.s001.jpg]

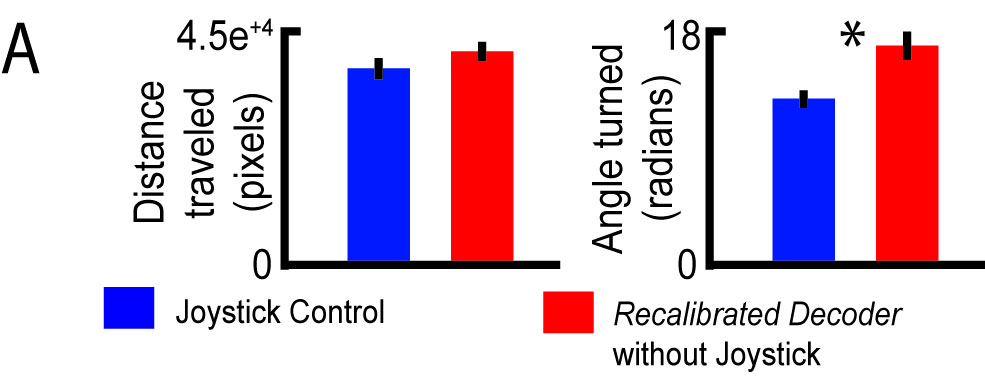

Supplement: S2 Fig — (Left) Distance travelled and (Right) amount turned for joystick and BMI control. Trajectories were tracked using an automatic object detection algorithm applied to videos taken during the trials. Error bars represent the standard error of the mean. Asterisks denote a significant difference (ANOVA, p<0.05). (JPG) [file pone.0165773.s002.jpg]
